# Metformin Preserves β-Cell Compensation in Insulin Secretion and Mass Expansion in Prediabetic Nile Rats

**DOI:** 10.3390/ijms22010421

**Published:** 2021-01-03

**Authors:** Hui Huang, Bradi R. Lorenz, Paula Horn Zelmanovitz, Catherine B. Chan

**Affiliations:** 1 Department of Physiology, University of Alberta, Edmonton, AB T6G 2H7, Canada; hhuang2@ualberta.ca (H.H.); blorenz@ualberta.ca (B.R.L.); 2 Department of Psychology, University of Alberta, Edmonton, AB T6G 2R3, Canada; zelmanov@ualberta.ca; 3 Department of Agricultural, Food and Nutritional Science, University of Alberta, Edmonton, AB T6G 2P5, Canada; 4 Li Ka Shing Center for Health Research Innovation, University of Alberta, Edmonton, AB T6G 2E1, Canada

**Keywords:** metformin, prediabetes, β-cell compensation, UPR, proliferation

## Abstract

Prediabetes is a high-risk condition for type 2 diabetes (T2D). Pancreatic β-cells adapt to impaired glucose regulation in prediabetes by increasing insulin secretion and β-cell mass expansion. In people with prediabetes, metformin has been shown to prevent prediabetes conversion to diabetes. However, emerging evidence indicates that metformin has negative effects on β-cell function and survival. Our previous study established the Nile rat (NR) as a model for prediabetes, recapitulating characteristics of human β-cell compensation in function and mass expansion. In this study, we investigated the action of metformin on β-cells in vivo and in vitro. A 7-week metformin treatment improved glucose tolerance by reducing hepatic glucose output and enhancing insulin secretion. Although high-dose metformin inhibited β-cell glucose-stimulated insulin secretion in vitro, stimulation of β-cell insulin secretion was preserved in metformin-treated NRs via an indirect mechanism. Moreover, β-cells in NRs receiving metformin exhibited increased endoplasmic reticulum (ER) chaperones and alleviated apoptotic unfold protein response (UPR) without changes in the expression of cell identity genes. Additionally, metformin did not suppress β-cell mass compensation or proliferation. Taken together, despite the conflicting role indicated by in vitro studies, administration of metformin does not exert a negative effect on β-cell function or cell mass and, instead, early metformin treatment may help protect β-cells from exhaustion and decompensation.

## 1. Introduction

Prediabetes, a high-risk condition for T2D, is defined by impaired glucose tolerance (IGT) or impaired fasting glucose (IFG) [1]. The prevalence of prediabetes is increasing; an estimated 470 million adults worldwide will have prediabetes by 2030, and up to 70% of the prediabetic population will progress to diabetes [2]. Much effort has been made to identify effective interventions to prevent or even reverse prediabetes conversion to T2D [3,4]. Data collected in the Diabetes Prevention Program (DPP) outcomes study suggests that, other than lifestyle intervention, metformin is a cost-effective pharmaceutical option to delay the progression of T2D in prediabetic subjects [4,5,6].

Metformin is a biguanide anti-diabetic medication that has been used to treat T2D for decades. It exerts its glucose-lowering effect by inhibiting hepatic glucose production and increasing glucose utilization [7]. Evidence from in vitro studies reveals that metformin protects β-cells from dysfunction and apoptosis against glucolipotoxicity [8,9,10,11]. However, given that metformin inhibits mitochondrial ATP production, which is essential for β-cell insulin secretion, metformin is also proposed to directly inhibit β-cell insulin secretion and survival [12,13,14]. With little evidence regarding in vivo metformin actions on β-cells during prediabetes, the role that metformin could play to advance or prevent the progression of β-cell dysfunction is not clear.

NR (*Arvicanthis niloticus*) is a wild rodent that develops T2D naturally when adapted to a laboratory setting and fed with a standard chow diet (Chow) [15]. Our previous study demonstrated that NR is also a model for prediabetes, recapitulating characteristics of human β-cell compensation, starting at the age of two months in response to insulin resistance [16]. This progression of T2D in NRs, as well as the changes in β-cell function, was prevented by providing a diet with lower caloric density and higher fiber content (Hfib) [16,17]. In the present study, taking advantage of the spontaneous β-cell adaptative response in the NR, we sought to investigate the long-term effect of metformin on glucose regulation, β-cell function, and survival in prediabetes.

## 2. Results

### 2.1. Early Treatment of Metformin Improves Glucose Tolerance and Insulin Secretion In Vivo in Newly Prediabetic NRs

**Figure 1:**
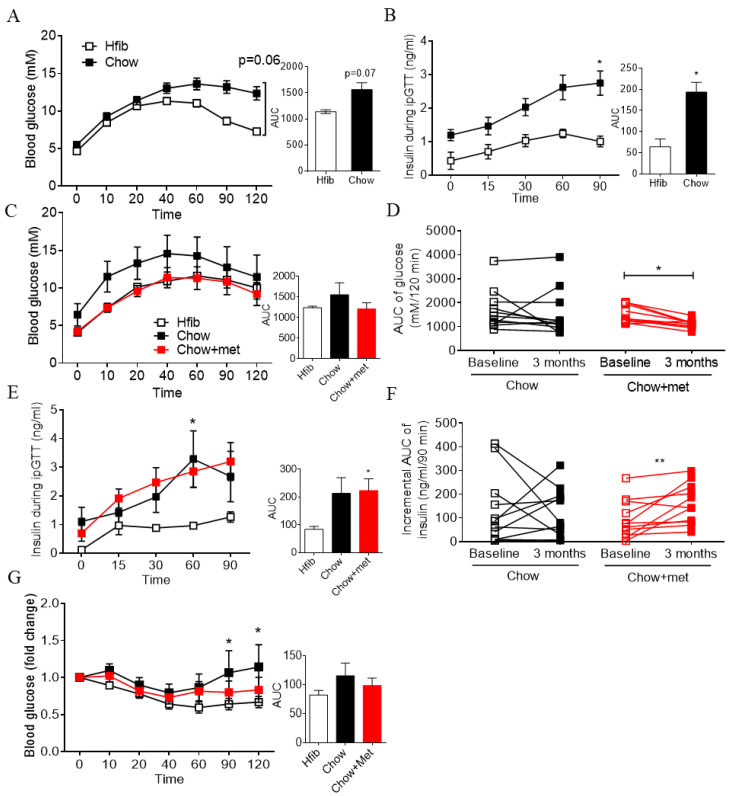
Glucose tolerance and insulin secretion was improved by metformin in prediabetes. (**A**–**F**): blood glucose (**A**,**C**) and insulin secretion (**B**,**F**) in ipGTT at baseline six weeks (**A**,**B**) or after treatment at three months (**C**–**F**); (**D**,**F**): comparisons of the area under the curve (AUC) of glucose (**D**) or insulin secretion (**F**) before and after treatment. (**G**): percentage change in blood glucose during ITT at three months. AUC data are presented as mean + SEM. *n* = 5 in Hfib; 11 in Chow and Chow + met group. For ipGTT, * *p* < 0.05 vs Hfib, ** *p* < 0.01 vs Hfib using two-way ANOVA or Kruskal–Wallis test (AUC) followed by post hoc multiple comparison tests. For comparison of AUC before and after treatment, * *p* < 0.05, ** *p* < 0.01 using Wilcoxon matched-pairs signed-rank test.

NRs exhibit overnutrition-induced euglycemic hyperinsulinemia at an early age [16]. To characterize the model used in this study, we first examined their glucose tolerance before treatment. In the intraperitoneal glucose tolerance test (ipGTT) at six weeks, NRs on the Chow diet displayed a moderate impairment in glucose tolerance (overall *p* < 0.05, Figure 1A) and a marked increase in insulin secretion (overall *p* < 0.001, Figure 1B), which confirms the presence of prediabetes in this model. After seven weeks of metformin treatment, Chow-fed NR displayed fasting hyperinsulinemia and hyperglucagonemia compared with Hfib NR (Supplementary Table S1). Compared with Chow, bodyweight, or hormone levels were not affected by metformin treatment (Supplementary Table S1). Despite that, approximately 10% of NRs in the Chow group exhibited IFG (fasting glucose over 5.6 mM), which was absent in Hfib or Chow+met group. For glucose regulation, Chow + met NRs showed comparable glucose tolerance to Hfib NRs (overall treatment effect *p* < 0.05, Figure 1C), a significant (*p* < 0.05) improvement vs baseline at six weeks, while Chow NRs had no change (*p* > 0.05) before and after (Figure 1D). Insulin secretion was not impaired by metformin (Figure 1E) but elevated compared to baseline at six weeks (Figure 1F). Moreover, the glycemic excursion of Chow-feds NRs in the intraperitoneal insulin tolerance test (ITT) was significantly different from the Hfib group in the glucose recovery phase (t = 90 and 120, overall treatment effect *p* < 0.05, Figure 1G), suggesting elevated hepatic glucose production exclusively in Chow NRs. 

### 2.2. Metformin Suppresses Hepatic Glucose Production and Expressions of Gluconeogenic Enzymes 

**Figure 2:**
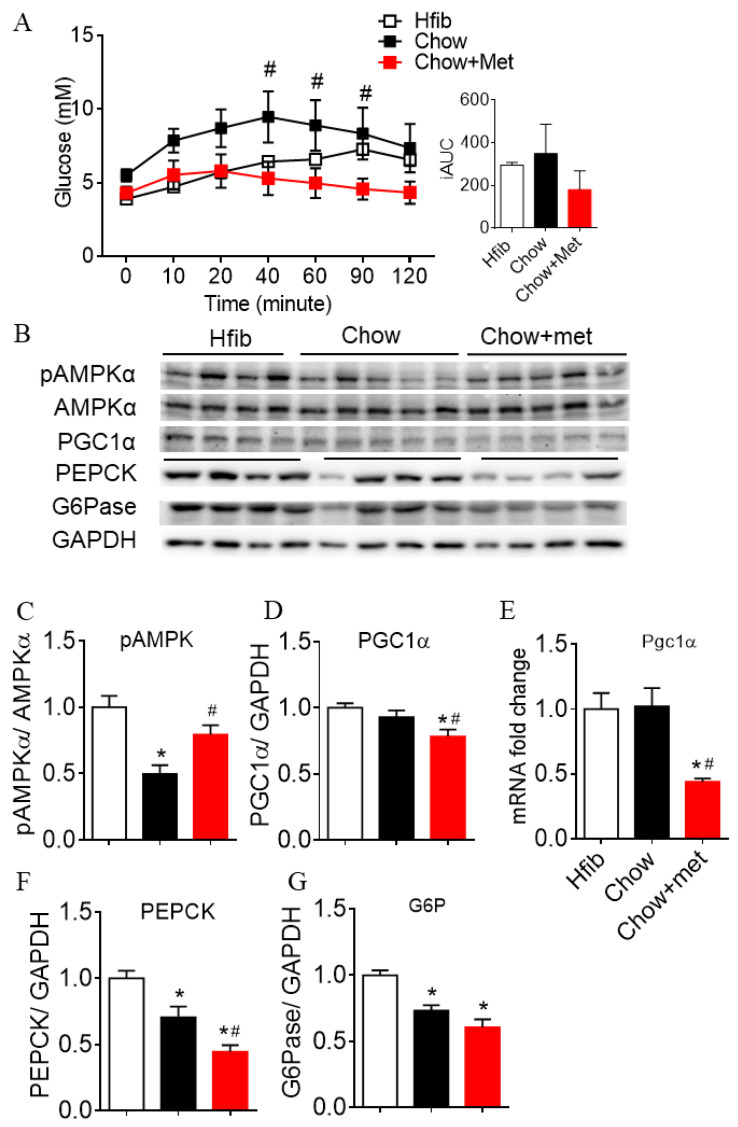
Metformin inhibited hepatic glucose production and glucogenic enzyme expression in the fed condition. (**A**): blood glucose and AUC in pyruvate tolerance test (PTT); (**B**): Representative images of liver protein; (**C**, **D**, **F**, and **G**): quantification of phospho-AMPK to AMPK (**C**), PCG1α (**D**), G6Pase (**F**), and PEPCK (**G**) relative to GAPDH; (**E**): gene transcription of PGC1α normalized to β-actin. Data are presented as mean + SEM. *n* = 4–6. * indicates *p* < 0.05 vs. Hfib, # *p* < 0.05 vs. Chow using the Kruskal–Wallis test followed by Dunn’s multiple comparison test.

The liver is the major target of metformin, the action of which reduces glucose production through regulating gluconeogenic gene expressions via AMP-activated protein kinase (AMPK) [18,19]. In line with this classic mechanism, we first showed that the glucose elicited by exogenous pyruvate in Chow + met NRs was reduced compared to Chow rats (overall treatment effect *p* < 0.05, Figure 2A), indicating lower hepatic gluconeogenesis with metformin [20,21]. Hepatic AMPK phosphorylation was significantly downregulated in Chow-fed NRs vs. Hfib, which was restored by metformin (Figure 2B,C). PPARγ coactivator 1α (PGC-1α) is a downstream transcriptional coactivator to the gluconeogenic enzymes phosphoenolpyruvate carboxykinase (PEPCK) and glucose 6-phosphatase (G6Pase) [22]. Here in this study, PGC1α and PEPCK were downregulated in Chow + met NRs compared to Hfib and Chow (Figure 2B,D–F). G6Pase abundance was altered by diet but not metformin treatment (Figure 2B,G).

### 2.3. Islet Glucose-Stimulated Insulin Secretion Is Preserved in Metformin-Treatment Prediabetic NRs

**Figure 3:**
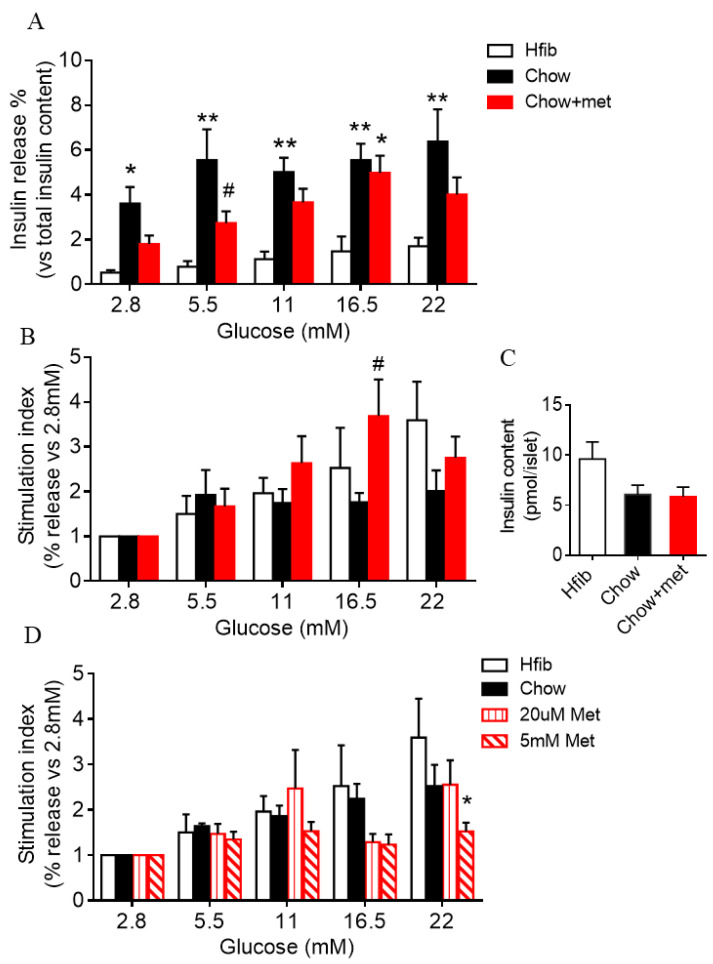
Islet glucose-stimulated insulin secretion (GSIS) was preserved in metformin-treatment NRs. (**A**,**B**): GSIS of islet isolated from NRs presented as percentage insulin release relative to islet insulin content (**A**) or baseline insulin secretion at 2.8 mM glucose (**B**); (**C**): average islet insulin content; (**D**): GSIS in fold change of islets treated with 20 µM or 5 mM metformin for 24 h; Data are presented as mean + SEM. *n* = 8–12 (**A**–**C**), 4–6 (**D**). * indicates *p* < 0.05 vs. Hfib, ** *p* < 0.01 vs. Hfib, # *p* < 0.05 vs. Chow using two-way ANOVA followed by post hoc multiple comparison tests; Iinsulin content was analyzed using the Kruskal–Wallis test followed by Dunn’s multiple comparisons tests.

Metformin is known to inhibit ATP synthesis, which could attenuate β-cell glucose-stimulated insulin secretion (GSIS) [12]. However, we observed enhanced insulin secretion in the GTT in metformin-treated NRs (Figure 1). To explore whether the islet secretory capacity is altered, insulin secretion was assessed in isolated islets challenged with basal (2.8–5.5 mM) and stimulated (11–22 mM) glucose. In line with previous data, Chow islets had a greater insulin secretion than Hfib (Figure 3A). Whereas Chow + met islets exhibited significantly increased stimulated secretion (16.5 mM, *p* < 0.05 vs. Hfib) but a more regulated basal secretion (5.5 mM) compared to Chow (Figure 3A). After normalized to basal secretion, the stimulation index of Chow islets was significantly lower than Hfib and Chow + met (Figure 3B). This suggests the unregulated GSIS phenotype in Chow is alleviated in Chow + met NRs. No difference was observed between Chow and Chow + met in islet insulin content (Figure 3C).

We then sought to test whether the improvement of islet function was via direct action of metformin on islets. To test that, islets isolated from prediabetic NRs were treated with metformin at 20 µM, a dose that mimics the plasma concentration of metformin [23], or at 5 mM as it is a concentration commonly used to inhibit β-cell insulin secretion in vitro [9,12]. Islets subjected to 24 h treatment with metformin showed no evident improvement in secretion with 20 µM metformin, while 5 mM metformin-treated islets had diminished insulin secretion, especially under stimulatory glucose conditions (Figure 3D).

### 2.4. β-Cell Compensation with Reduced Apoptotic Unfolded Protein Response in NRs Treated with Metformin

**Figure 4:**
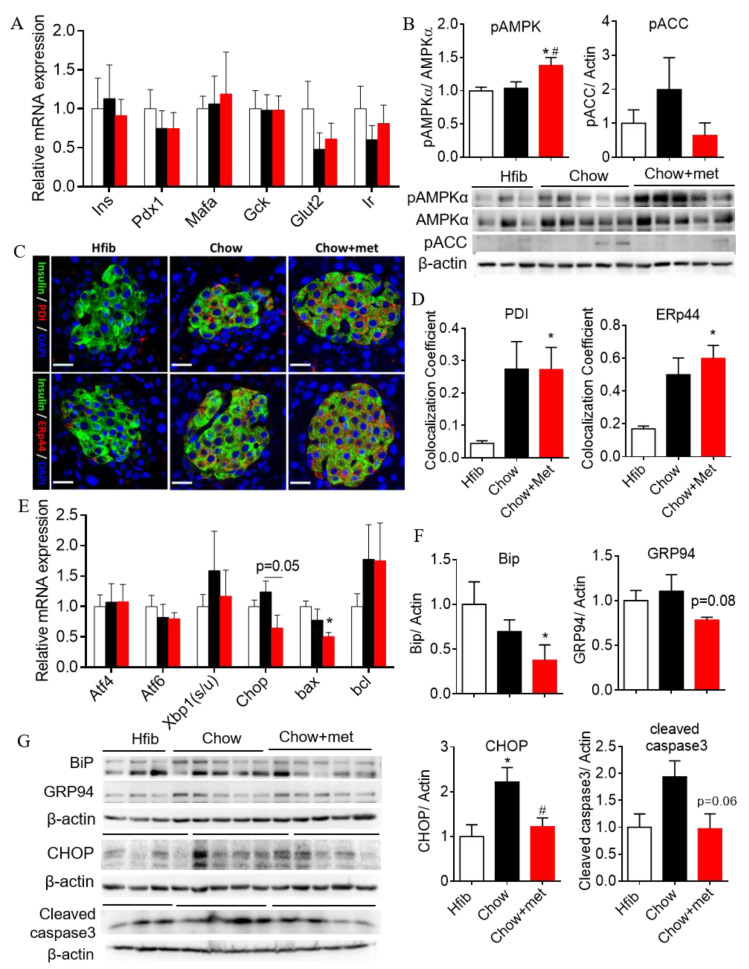
Assessment of β-cell identity gene and unfolded protein response (UPR) markers in islets. (**A**): mRNA of keys genes to β-cells in islets; (**B**): quantification of phosphorylation of AMPK and acetyl-CoA carboxylase (ACC) in islets. (**C**): a confocal image of islets showing ER chaperones colocalized with insulin; (**D**): colocalization coefficient of ER chaperone with insulin; (**E**): mRNA expression UPR transcription factors. Data are presented as means ± SEM; (**F**): Protein abundance quantification of BiP, GRP94, CHOP, and cleaved caspase 3 in islets; (**G**): Representative images of BiP, GRP94, CHOP, and cleaved caspase 3 blots. *n* = 4–5. * indicates *p* < 0.05 vs. Hfib, # *p* < 0.05 vs. Chow using the Kruskal–Wallis test followed by Dunn’s multiple comparison test. Scale bar = 20 μm.

To gain insight into the molecular mechanisms that may play a role in altering islet function during diabetes progression and metformin treatment, we then looked at the expression of genes critical to β-cell function and survival [24,25]. No significant change in gene transcription of selected markers was observed in isolated islets between groups (Figure 4A). Metformin has been shown to active insulin receptor [26]. By Western blot, we did observe an increase in the abundance of insulin receptor (IR) in Chow + met islets (Supplementary Figure S1) but failed to detect phosphorylated IR or insulin receptor substrate. Metformin also affects lipid metabolism through inhibiting acetyl-CoA carboxylase (ACC), which is a key enzyme in fatty acid synthesis [18]. Here we showed increased AMPK phosphorylation exclusively in Chow + met islets (Figure 4B), but the decrease in ACC phosphorylation was not significant vs. Chow (Figure 4B).

Our previous study showed that ER chaperones that facilitate insulin synthesis are upregulated in β-cells during insulin secretory compensation as a mechanism of adaptive UPR [16]. In Chow + met islets, insulin-colocalized with protein disulfide isomerase (PDI) and ER-resident protein 44 (ERp44) were significantly increased (Figure 4C,D). While pro-apoptotic UPR marker binding immunoglobulin protein (BiP), C/EBP homologous protein (CHOP), Bax (Bcl-2-associated X protein), as well as apoptotic marker cleaved caspase 3 were downregulated in Chow+met islets (Figure 4E–G), indicating alleviated apoptotic UPR with metformin.

### 2.5. β-Cell Mass and Regeneration Does Not Change with Metformin

**Figure 5:**
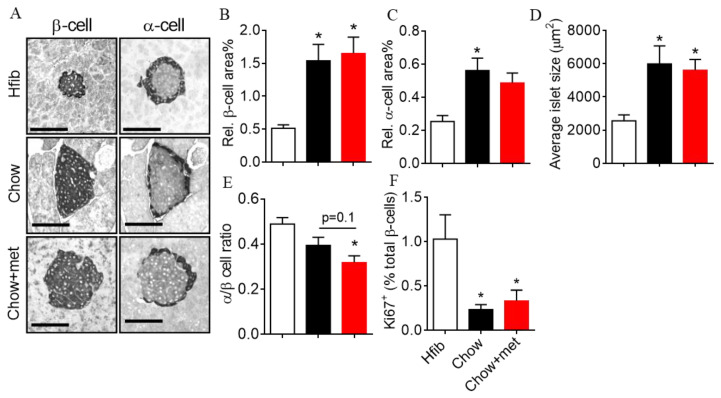
Pancreatic α-/β-cell area and β-cell proliferation. (**A**,**F**): representative images of insulin and glucagon staining; (**B**,**C**): relative β- (**B**) and α-cell (**C**) area to the total pancreas area; (**D**): average islet size; (**E**): Ratio of α- to β-cell area; (**F**): Percentage of the number of proliferating β-cell to the total number of β-cell. *n* = 5 in Hfib group; *n* = 11 in Chow and Chow-met groups. * *p* < 0.05 using the Kruskal–Wallis test followed by Dunn’s multiple comparisons tests. Scale bar = 100 μm.

In addition to its actions on cell function, recent diabetes and cancer research reveal an inhibitory effect of metformin on cell proliferation under physiological conditions [27,28]. Thus, we investigated the islet morphology and β-cell proliferation in metformin-treated NRs. The β-cell area increased by 3-fold in Chow-fed NRs compared to Hfib, but was not different between Chow and Chow + met treatments (Figure 5A,B). The α-cell area and average islet size were also elevated, with a moderately reduced α/β ratio (Figure 5A,C–E). Consistent with the previous results regarding β-cell proliferation, β-cells in Chow NRs demonstrate a lower incidence of proliferation as indicated by ki67 positive nuclei in insulin-positive cells, notwithstanding the larger cell area (Figure 5E). There is no difference in individual cell area or proliferation between metformin-treated and untreated groups (Figure 5F).

## 3. Discussion

### 3.1. The Effect of Metformin Dose in Glucose Regulation

Metformin has been suggested as a treatment for prediabetes and insulin resistance in numerous studies [4,27,29,30,31,32]. Interestingly, most animal trials reported complete reversal of insulin resistance, whereas clinical trials showed a far less evident outcome with metformin [4,32,33]. The clinically effective dose of metformin for diabetes prevention is 850 mg twice daily, which is equivalent to 20–50 mg/kg body weight [4]. In animal studies, metformin doses vary from 50 to 200 mg/kg depending on the species and diabetic condition of the model [30,31]. A recent study revealed that a low dose of metformin (10 mg/kg) mimics the effect of caloric restriction in mice, improving insulin sensitivity with aging and preventing the onset of metabolic syndrome; whereas a higher dose (100 mg/kg) shortens the mean lifespan likely due to renal failure [34]. Given that the effect of metformin is dose-dependent [35], the different efficacy may be a result of the 10-fold higher dose used in animal studies, which is not applicable in clinical trials due to consideration of side effects [36]. Herein, the use of metformin at a relatively low dose could represent a more realistic replication of the clinical situation. After seven weeks of metformin treatment, glucose tolerance was improved concomitant with enhanced insulin secretion. Although metformin did not reverse the prediabetes, it prevented the decompensation of Chow NRs by reducing hepatic glucose production, sustaining insulin secretion compensation, and protecting β-cells against dysfunction. 

Insulin resistance in the liver is a major contributor to the pathogenesis of prediabetes [37,38]. Dysfunctional mitochondria and disrupted insulin signaling due to excessive lipid metabolism lead to uncontrolled hepatic glucose production [39,40], while metformin inhibits mitochondrial function, thereby activating AMPK, which switches off the gluconeogenic pathway as shown in our results [18,41,42]. Metformin also improves insulin signaling and counteracts the action of glucagon in the liver [43,44]. In fasted NRs, protein kinase A (PKA) was activated in response to the marginal rise in circulating glucagon in Chow + met NRs but failed to induce PEPCK and G6Pase, which may be neutralized by AMPK activation with metformin (Supplementary Figure S2). Of note, healthy NRs fed on the Hfib diet demonstrate a high abundance of G6Pase and PEPCK throughout the study, reflecting an overall enhanced hepatic glucose production that is likely advantageous in their native environment but which could be a risk factor for glucose intolerance when switching to a high-energy and high refined carbohydrate diet like Chow.

In clinical trials, decreased food intake and weight loss are related to the glucose-lowering effect of metformin, the mechanism of which is linked to decreased appetite [45]. However, low dose metformin did not change the bodyweight of NRs, which may be attributed to a lack of suppression of appetite. However, due to the feral behavior of NRs, we could not track their food intake, which is a limitation of the study.

### 3.2. Metformin Actions on β-Cell Function and Possible Mechanisms

In human islets, the effect of metformin on β-cells function is controversial. Since metformin is the f in human islets, the effect of metformin on β-cell function is controversial. Because metformin is the first-line drug for T2D, whether it reverses defects in islet function in prediabetic and T2D human patients is a trending topic. Loss of GSIS after exposure to high glucose is termed ‘glucotoxicity’. Early studies in healthy human islets found that 15 µM metformin for 24h blocked the development of glucotoxicity [8,46]. In addition, Lupi et al. also reported a protective effect of metformin on GSIS inhuman islets treated with 2mM free fatty acid (FFA) [47]. Moreover, islets from T2D donors incubated with metformin for 24h had partially restored islet insulin content and GSIS as well as reduced oxidative stress and apoptosis [10]. On the contrary, inhibition of insulin secretion by metformin has been reported in both rodent and human islets [12,48,49]. After a 16h incubation with 1 mM metformin, GSIS from human islets was significantly decreased [12]. In rodent islets, a high dose of metformin inhibited insulin biosynthesis and cell survival [48,49].

When evaluating the evidence regarding metformin on islet function, it is critical to compare the metformin concentration used in the in vitro setting to achieve therapeutic plasma metformin concentrations. According to the pharmacokinetics of metformin in humans, the plasma metformin peaks at 3 µg/mL (18 µM) after oral administration of a 1.5 g dose and is maintained at concentrations lower than 0.2 µg/mL [50,51]. In rodents, the peak is 18 µg/mL (108 µM) after a 200 mg dose [23], both of which are much lower than 1 mM. Therefore, it seems unconvincing that the plasma concentrations of metformin in vivo exert inhibitory actions on islet function. Despite that, it has been recognized that the cellular metformin concentration could be much higher than in circulation as it tends to accumulate in mitochondria [42]. In the current study, Chow islets exhibited unregulated basal insulin secretion and decreased GSIS, which are features of β-cell decompensation [52,53], whereas islets isolated from metformin-treated NRs (Chow + met) exhibited improved GSIS with alleviated hypersecretion at basal glucose and improved GSIS. These results support the notion that metformin protects islets from dysfunction. Besides, metformin in vivo did not alter β-cell identity or suppress insulin expression. Despite that, a 24h incubation with 20 µM metformin in vitro did not restore impaired GSIS as reported previously [10]. Metformin at 5mM mildly suppressed insulin secretion, which, however, was not seen in vivo. The results emphasize the necessity of carefully evaluating and comparing findings from in vitro islets in in vivo studies.

The classic mechanism of the anti-diabetic actions exerted by metformin is through inhibiting the mitochondrial respiratory chain complex, which leads to a reduction of ATP and increases in the AMP/ATP ratio [41,42]. Subsequent phosphorylation of AMPK activates a serial of downstream actions [18]. However, ATP production is critical to insulin secretion because increased ATP/ADP closes the ATP-sensitive potassium channel and initiates insulin granule exocytosis [54,55]. Evidence from human islets showed that 24h treatment with 15 µM metformin does not decrease the ATP/ADP ratio of islets under normal conditions and restores the impaired ATP/ADP ratio caused by glucotoxicity [56]. Also, metformin prevents the high glucose-induced ultrastructural abnormalities of mitochondria and ER in human islets [56]. In a lipotoxicity islet model, 48h FFA-treated islets exhibited a hypersecretion pattern during GSIS, which decreased significantly at day seven, and this was prevented by co-incubation with 25 µM metformin [57]. In the dynamics of islet function, metformin showed a mild inhibitory effect on mitochondrial oxidation and activation of AMPK [57]. In this study, we showed enhanced AMPK phosphorylation in Chow + met islets, suggesting AMPK may be involved in metformin action. However, the exact role of AMPK in insulin secretion is not clear as the activation of AMPK by high-dose metformin or overexpression in human islets impairs insulin secretion [49]. Also, decreased GSIS by a high-dose of metformin can be reversed by 25 mM glucose, albeit with the inhibition of mitochondrial oxidation in INS1 cells [14]. Therefore, how mitochondrial oxidation and AMPK is involved in metformin action remains to be clarified. 

In addition to AMPK, evidence from FFA-treated islets suggests that metformin alleviates oxidative stress [9,11]. In rodent islets, metformin inhibits FFA-induced oleate oxidation, restoring GSIS and reducing reactive oxygen species (ROS) and inducible nitric oxide synthase (iNOS) [9,11]. Excessive ROS and iNOS induce oxidative stress, mitochondrial damage, impaired GSIS, and apoptosis [58,59,60]. Despite the AMPK activation in Chow+met NR islets, we did not observe a significant increase in phosphorylated ACC, the form of ACC that catalyzes fatty acid synthesis [18]. More evidence is required from human islets or in vivo studies to test the hypothesis that FFA oxidation and oxidative stress are involved in metformin mechanisms.

Another mechanism of metformin in islets is decreased ER stress [57]. ER stress plays a dual role in islet dysfunction [61]. With increased insulin demand, adaptive UPR is activated by the accumulation of unfolded proinsulin in the ER, leading to increased expression of ER chaperones and folding enzymes [62,63,64]. Chronic ER stress, on the other hand, exerts apoptotic UPR and cell apoptosis [61]. A recent finding in human islets suggested that the presence of 25 µM metformin ameliorated apoptotic UPR through decreased CHOP and caspase 3 [57]. Our previous study revealed that ER chaperones increase concomitantly with β-cell compensation [16]. Here, with metformin, PDI and ERp44 in islets were maintained, while expression of apoptotic markers including CHOP, Bax, and cleaved caspase 3 was reduced, suggesting a shift of apoptotic UPR to adaptive UPR with metformin. BiP is an upstream effector that senses the unfolded protein perturbation in ER [65,66]. The reduced BiP in Chow + met islets suggests alleviated stress and restoration of a healthier ER environment. Taken together, metformin protects islet function partially via reduced ER stress.

### 3.3. Metformin Actions on β-Cell Mass and Survival

In addition to islet function, another concern for using metformin to treat prediabetes is its ability to inhibit cell proliferation, as shown in cancer research [28,67]. This could lead to suppressed β-cell mass compensation during prediabetes. Our results showed no difference in β-cell mass in prediabetic NRs with or without metformin, both of which had increased β-cell mass compared to healthy controls. On the contrary, Tajima et al. found that metformin abolishes β-cell mass expansion in obesity-induced prediabetic mice together with a marked reduction in insulin secretion [27]. As β-cell compensation is tightly associated with glucose and insulin concentrations [68,69], the absent mass expansion could be an indirect effect of metformin actions on glucose metabolism. Indeed, Wyett et al. reported no inhibitory effect of metformin on β-cell during development or regeneration after β-cell ablation [70]. Although some in vitro evidence shows that metformin suppresses proliferation and induces apoptosis [27,49,71], the decreased cleaved caspase 3 in Chow + met islets, as well as the maintained β-cell mass, indicated no pro-apoptotic activity of metformin.

In summary, the present study demonstrates that metformin treatment of insulin-resistant, prediabetic NRs improves glucose tolerance, insulin secretion, and overall insulin sensitivity, which is accompanied by downregulated expressions of hepatic glucogenic enzymes. The observation of preserved β-cell secretion capacity with increased adaptive UPR and reduced apoptotic markers indicates a protective role for metformin in β-cell function. Additionally, metformin does not suppress β-cell proliferation or β-cell mass expansion. Taken together, despite the conflicting role indicated by in vitro studies, metformin does not exert a negative effect on β-cell function or cell mass, and instead, early metformin treatment may help protect β-cell from exhaustion in insulin secretion and apoptosis.

## 4. Methods and Materials

### 4.1. Animals

This study was approved by the University of Alberta Animal Care and Use Committee (#00000328, 24 January 2018). All animal experiments were performed following the Canadian Council on Animal Care Guidelines. The NRs were obtained from the colony maintained at the University of Alberta and housed under 14 h/10 h-light/dark cycle. At three weeks, NRs were weaned to a chow diet (Chow, Prolab RMH 2000, LabDiet) or a high fiber, low energy diet (Hfib, Mazuri Chinchilla, 5M01, PMI Nutrition International) as previously described [16,17].

### 4.2. Drug Administration

Animal fed on chow diet in each litter were randomized into Chow or Chow+met group to avoid litter effect. Metformin (Millipore Sigma, St. Louis, MA, USA) was administered at 20 mg/kg body weight/day in drinking water beginning three weeks after weaning. Water intake was measured weekly before and during the treatment. Metformin concentration in drinking water was adjusted based on animal water intake and body weight, if necessary.

### 4.3. Glucose, Insulin, and Pyruvate Tolerance Tests

Animals in Hfib, Chow, and Chow + met were subject to intraperitoneal ipGTT at six weeks and three months of age to assess the baseline and changes in glucose metabolism after treatment. The intraperitoneal ITT or PTT tests were used to estimate insulin responsiveness and hepatic gluconeogenesis in vivo. ipGTT, PTT, and ITT were performed on animals following overnight or 4 h fasting. Due to the feral behavior of NRs, all tolerance tests were done in a surgery room, and animals were anesthetized with isoflurane (Millipore Sigma) during procedures. ipGTT and ITT were measured by the method described [16]. For PTT, pyruvate was intraperitoneally injected at a dose of 2 g/kg body weight. Blood glucose was determined before and 10, 20, 40, 60, 90, 120 min after injection using a CONTOUR NEXT blood glucose monitoring system (Bayer Inc., Mississauga, ON, Canada). All standard operating procedures were approved by the University of Alberta Animal Care and Use Committee. Animals that did not recover from anesthesia in 30 min or had blood glucose lower than 2 mM during experiments were excluded from the study. The in vivo experiments were not done blindly as metformin-treated animals were regarded as chemical hazard-treated, which requires additional personal protective equipment.

### 4.4. Tissue Collection

Animals were euthanized following overnight fasting or a 16 h fasting and 4 h refeeding protocol to allow measurement of the metabolic status and the expression of hepatic gluconeogenic enzymes under fasting and fed conditions, respectively. Fasting blood glucose was measured before administration of Euthanyl. To avoid degradation of liver proteins, a lobe of the liver was harvested and snap-frozen immediately after animals achieved a surgical plane of anesthesia without damaging the common bile duct for pancreas perfusion. Blood samples were then collected via cardiac puncture for measurement of insulin, glucagon, and glucagon-like-peptide 1 (GLP-1). DPP-IV inhibitor (10 µmol/L, Millipore, Billerica, MA, USA) was added to blood collected for GLP-1 measurement. Insulin, glucagon, and GLP-1 concentrations were determined by ELISA (Insulin, Crystal Chem Inc., Downers Grove, IL, USA; glucagon, ALPCO Diagnostics Inc., Salem, NH; GLP-1, Meso Scale Discovery, Rockville, MD, USA). The pancreas tail was clamped and collected from fasted animals and fixed in 10% Neutral Buffered Formalin for immunohistochemical (IHC) examination. Islet isolation and calculation of body mass index (BMI), insulin sensitivity index (ISI), homeostatic model assessment of insulin resistance (HOMA-IR), and the homeostatic model assessment of β-cell function (HOMA-B) were as described previously [16].

### 4.5. In Vitro Metformin Treatment and Glucose-Stimulated Insulin Secretion

To measure the effects of sub-acute, direct, in vitro metformin treatment on islet function, isolated islets from Chow NRs at the age of three to four months were cultured in Dulbecco’s modified Eagle’s medium (DMEM) containing 8.3 mM glucose, 10% bovine serum with or without 20 µmol/L or 5 mmol/L metformin for 24 h. Metformin was supplemented in the medium used in the GSIS assay as well. To examine the effects of chronic, in vivo metformin treatment, islets from the metformin-treated group were cultured in the absence of exogenous metformin. Control islets for both sub-acute and chronic metformin experiments were from Chow NRs.

To assess insulin secretion, three isolated islets were incubated in 1 mL of DMEM supplemented with concentrations of glucose of 2.8, 5.5, 11.0, 16.5, or 22 mM for 90 min. The supernatant was transferred into new tubes, and the pellets were lysed using 1 mL of 3% acetic acid. Insulin in the supernatant and the pellet was determined by insulin radioimmunoassay. Absolute insulin secretion indicates the amount of insulin secreted per islet during the 90-min incubation. Insulin release % was insulin secretion normalized by the corresponding islet insulin content. The stimulation index reflects the ability of islet in insulin secretion at high glucose concentrations and was calculated as the ratio of stimulated (X.X glucose) to basal secretion at 2.8 mM glucose. 

### 4.6. Immunofluorescent Microscopy

Pancreatic tails dissected from NRs were fixed in formalin overnight, sectioned into 5 µm slices, and mounted on glass slides (Superfrost Plus Microscope Slides, Fisherbrand). Antibodies used in immunohistochemistry- or immunofluorescent -staining were listed in Supplementary Table S1. Insulin- and glucagon- stained sections were scanned using the Leica DM 6000B platform and analyzed with Image J 6.0. The α-/β-cell areas % was calculated by taking the glucagon- or insulin-positive area divided by total pancreatic section area. Ki67/insulin co-stained sectioned were imaged with Zeiss Axio Imager. Ki67 positive β-cell% was evaluated by the number of ki67/ insulin double-positive cells divided by the total number of insulin-positive cells. ER protein/insulin co-stained images were obtained using Quorum’s WaveFX confocal system (Quorum Technologies) and analyzed with Volocity 6.0 (PerkinElmer) as previously described [17].

### 4.7. Semi-Quantitative Real-Time PCR

Total RNA was extracted from islets (>30 islets) or frozen liver (50–100 mg) using TRIzol reagent (Invitrogen, Carlsbad, CA, USA). We used 1 µg of RNA to synthesize cDNA using QuantiTect reverse transcription kit (QIAGEN, Mississauga, ON, Canada). Because the NR genome has not been sequenced, primers were designed from regions conserved between rat and mouse (Supplementary Table S2). Real-time PCR was performed using an SYBR green qPCR Mastermix (Quanta Biosciences, Gaithersburg, MD, USA) and Rotor Gene 6000 Real-time PCR machine (Corbett Research). The qPCR specificity was confirmed by agarose gel electrophoresis and efficiency of 90%–110%. The data were analyzed using the delta Ct method [72]. Target gene expression was normalized to β-actin.

### 4.8. Western Blot Analysis

Snap-frozen liver (up to 50 mg) was homogenized in 0.5 mL of RIPA buffer supplemented with proteinase inhibitor cocktail, aprotinin (2 µg/mL), sodium fluoride (5 mmol/L), sodium orthovanadate (1 mmol/L) and PMSF (1 mmol/L). All reagents were from Millipore Sigma. The concentration of protein extracts was determined by bicinchoninic acid assay, and proteins were diluted to a final concentration of 2 μg/μL with RIPA buffer and SDS loading buffer. About 50 μg of protein was loaded for each sample. Proteins were separated on 10% SDS-PAGE gels and transferred to nitrocellulose membranes. Membranes were blocked for 1 h with 5% skim milk or 3% BSA in TBS (20 mM Tris, 137 mM NaCl, pH 7.6) with 0.1% Tween-20 and probed with antibodies (Supplementary Table S1). Blots were developed using ECL (Thermo Fisher Scientific) and imaged with a Bio-Rad ChemiDoc MP imaging system. Antibody detecting phosphorylated protein was stripped to enable detection of the corresponding total protein on the same blot.

### 4.9. Statistical Analyses 

For all experiments, data were expressed as the mean ± SEM and statistically analyzed with GraphPad Prism version 6.0. Outlier identified by ROUT was removed from the analysis. One-way or two-way analysis of variance was used, with repeated measures when appropriate, followed by posthoc multiple comparisons if significance was reached. Differences were considered significant at *p* < 0.05.

## Data Availability

The data presented in this study are available in “Metformin Preserves β-Cell Com-pensation in Insulin Secretion and Mass Expansion in Prediabetic Nile Rats.” and the supplementary material.

## Supplementary Materials

The following are available online at https://www.mdpi.com/1422-0067/22/1/421/s1, Figure S1: Protein abundance of GLUT2 (A) and IR (B) in isolated islets; Figure S2: Analysis of hepatic AMPK pathway in the fasting condition; Table S1: Metabolic profile of NRs treated with metformin; Table S2: Antibodies and dilutions used in western blot and immunofluorescent microscopy; Table S3: qPCR primers and annealing temperatures.

## References

[1] Punthakee Z., Goldenberg R., Katz P. (2018). Definition, Classification and Diagnosis of Diabetes, Prediabetes and Metabolic Syndrome. Can. J. Diabetes.

[2] Tabák A.G., Herder C., Rathmann W., Brunner E.J., Kivimäki M. (2012). Prediabetes: A high-risk state for diabetes development. Lancet.

[3] Knowler W.C., Narayan K.M., Hanson R.L., Nelson R.G., Bennett P.H., Tuomilehto J., Scherstén B., Pettitt D.J. (1995). Preventing non-insulin-dependent diabetes. Diabetes.

[4] Knowler W.C., Barrett-Connor E., Fowler S.E., Hamman R.F., Lachin J.M., Walker E.A., Nathan D.M. (2002). Diabetes Prevention Program Research Group Reduction in the incidence of type 2 diabetes with lifestyle intervention or metformin. N. Engl. J. Med..

[5] (2015). Diabetes Prevention Program Research Group Long-term effects of lifestyle intervention or metformin on diabetes development and microvascular complications over 15-year follow-up: The Diabetes Prevention Program Outcomes Study. Lancet Diabetes Endocrinol..

[6] Aroda V.R., Knowler W.C., Crandall J.P., Perreault L., Edelstein S.L., Jeffries S.L., Molitch M.E., Pi-Sunyer X., Darwin C., Heckman-Stoddard B.M. (2017). Metformin for diabetes prevention: Insights gained from the Diabetes Prevention Program/Diabetes Prevention Program Outcomes Study. Diabetologia.

[7] Hundal R.S., Krssak M., Dufour S., Laurent D., Lebon V., Chandramouli V., Inzucchi S.E., Schumann W.C., Petersen K.F., Landau B.R. (2000). Mechanism by which metformin reduces glucose production in type 2 diabetes. Diabetes.

[8] Lupi R., Del Guerra S., Tellini C., Giannarelli R., Coppelli A., Lorenzetti M., Carmellini M., Mosca F., Navalesi R., Marchetti P. (1999). The biguanide compound metformin prevents desensitization of human pancreatic islets induced by high glucose. Eur. J. Pharmacol..

[9] Patanè G., Piro S., Rabuazzo A.M., Anello M., Vigneri R., Purrello F. (2000). Metformin restores insulin secretion altered by chronic exposure to free fatty acids or high glucose: A direct metformin effect on pancreatic beta-cells. Diabetes.

[10] Marchetti P., Del Guerra S., Marselli L., Lupi R., Masini M., Pollera M., Bugliani M., Boggi U., Vistoli F., Mosca F. (2004). Pancreatic islets from type 2 diabetic patients have functional defects and increased apoptosis that are ameliorated by metformin. J. Clin. Endocrinol. Metab..

[11] Piro S., Rabuazzo A.M., Renis M., Purrello F. (2012). Effects of metformin on oxidative stress, adenine nucleotides balance, and glucose-induced insulin release impaired by chronic free fatty acids exposure in rat pancreatic islets. J. Endocrinol. Investig..

[12] Leclerc I., Woltersdorf W.W., da Silva Xavier G., Rowe R.L., Cross S.E., Korbutt G.S., Rajotte R.V., Smith R., Rutter G.A. (2004). Metformin, but not leptin, regulates AMP-activated protein kinase in pancreatic islets: Impact on glucose-stimulated insulin secretion. Am. J. Physiol. Endocrinol. Metab..

[13] Lamontagne J., Pepin E., Peyot M.-L., Joly E., Ruderman N.B., Poitout V., Madiraju S.R.M., Nolan C.J., Prentki M. (2009). Pioglitazone acutely reduces insulin secretion and causes metabolic deceleration of the pancreatic beta-cell at submaximal glucose concentrations. Endocrinology.

[14] Lamontagne J., Al-Mass A., Nolan C.J., Corkey B.E., Madiraju S.R.M., Joly E., Prentki M. (2017). Identification of the signals for glucose-induced insulin secretion in INS1 (832/13) β-cells using metformin-induced metabolic deceleration as a model. J. Biol. Chem..

[15] Chaabo F., Pronczuk A., Maslova E., Hayes K. (2010). Nutritional correlates and dynamics of diabetes in the Nile rat (Arvicanthis niloticus): A novel model for diet-induced type 2 diabetes and the metabolic syndrome. Nutr. Metab..

[16] Huang H., Yang K., Wang R., Han W.H., Kuny S., Zelmanovitz P.H., Sauvé Y., Chan C.B. (2019). β-Cell compensation concomitant with adaptive endoplasmic reticulum stress and β-cell neogenesis in a diet-induced type 2 diabetes model. Appl. Physiol. Nutr. Metab..

[17] Yang K., Gotzmann J., Kuny S., Huang H., Sauve Y., Chan C. (2016). Five stages of progressive beta-cell dysfunction in the laboratory Nile rat model of type 2 diabetes. J. Endocrinol..

[18] Zhou G., Myers R., Li Y., Chen Y., Shen X., Fenyk-Melody J., Wu M., Ventre J., Doebber T., Fujii N. (2001). Role of AMP-activated protein kinase in mechanism of metformin action. J. Clin. Investig..

[19] He L., Sabet A., Djedjos S., Miller R., Sun X., Hussain M.A., Radovick S., Wondisford F.E. (2009). Metformin and insulin suppress hepatic gluconeogenesis through phosphorylation of CREB binding protein. Cell.

[20] Calabuig-Navarro V., Yamauchi J., Lee S., Zhang T., Liu Y.-Z., Sadlek K., Coudriet G.M., Piganelli J.D., Jiang C.-L., Miller R. (2015). Forkhead Box O6 (FoxO6) Depletion Attenuates Hepatic Gluconeogenesis and Protects against Fat-induced Glucose Disorder in Mice. J. Biol. Chem..

[21] Hughey C.C., Wasserman D.H., Lee-Young R.S., Lantier L. (2014). Approach to assessing determinants of glucose homeostasis in the conscious mouse. Mamm. Genome.

[22] Koo S.-H., Flechner L., Qi L., Zhang X., Screaton R.A., Jeffries S., Hedrick S., Xu W., Boussouar F., Brindle P. (2005). The CREB coactivator TORC2 is a key regulator of fasting glucose metabolism. Nature.

[23] Choi Y.H., Kim S.G., Lee M.G. (2006). Dose-independent pharmacokinetics of metformin in rats: Hepatic and gastrointestinal first-pass effects. J. Pharm. Sci..

[24] Rutter G.A., Pullen T.J., Hodson D.J., Martinez-Sanchez A. (2015). Pancreatic β-cell identity, glucose sensing and the control of insulin secretion. Biochem. J..

[25] Marchetti P., Bugliani M., De Tata V., Suleiman M., Marselli L. (2017). Pancreatic Beta Cell Identity in Humans and the Role of Type 2 Diabetes. Front. Cell Dev. Biol..

[26] Gunton J.E., Delhanty P.J.D., Takahashi S.-I., Baxter R.C. (2003). Metformin rapidly increases insulin receptor activation in human liver and signals preferentially through insulin-receptor substrate-2. J. Clin. Endocrinol. Metab..

[27] Tajima K., Shirakawa J., Okuyama T., Kyohara M., Yamazaki S., Togashi Y., Terauchi Y. (2017). Effects of metformin on compensatory pancreatic β-cell hyperplasia in mice fed a high-fat diet. Am. J. Physiol. Endocrinol. Metab..

[28] Lord S.R., Cheng W.-C., Liu D., Gaude E., Haider S., Metcalf T., Patel N., Teoh E.J., Gleeson F., Bradley K. (2018). Integrated Pharmacodynamic Analysis Identifies Two Metabolic Adaption Pathways to Metformin in Breast Cancer. Cell Metab..

[29] Knowler W.C., Fowler S.E., Hamman R.F., Christophi C.A., Hoffman H.J., Brenneman A.T., Brown-Friday J.O., Goldberg R., Venditti E., Nathan D.M. (2009). 10-year follow-up of diabetes incidence and weight loss in the Diabetes Prevention Program Outcomes Study. Lancet.

[30] Linden M.A., Lopez K.T., Fletcher J.A., Morris E.M., Meers G.M., Siddique S., Laughlin M.H., Sowers J.R., Thyfault J.P., Ibdah J.A. (2015). Combining metformin therapy with caloric restriction for the management of type 2 diabetes and nonalcoholic fatty liver disease in obese rats. Appl. Physiol. Nutr. Metab..

[31] Wang N., Zhang J., Wu Y., Liu J., Liu L., Guo X. (2016). Metformin improves lipid metabolism disorders through reducing the expression of microsomal triglyceride transfer protein in OLETF rats. Diabetes Res. Clin. Pract..

[32] Lentferink Y.E., van der Aa M.P., van Mill E.G.A.H., Knibbe C.A.J., van der Vorst M.M.J. (2018). Long-term metformin treatment in adolescents with obesity and insulin resistance, results of an open label extension study. Nutr. Diabetes.

[33] Turner R.C., Cull C.A., Frighi V., Holman R.R. (1999). Glycemic control with diet, sulfonylurea, metformin, or insulin in patients with type 2 diabetes mellitus: Progressive requirement for multiple therapies (UKPDS 49). UK Prospective Diabetes Study (UKPDS) Group. JAMA.

[34] Martin-Montalvo A., Mercken E.M., Mitchell S.J., Palacios H.H., Mote P.L., Scheibye-Knudsen M., Gomes A.P., Ward T.M., Minor R.K., Blouin M. (2013). Metformin improves healthspan and lifespan in mice. Nat. Commun..

[35] Hirst J.A., Farmer A.J., Ali R., Roberts N.W., Stevens R.J. (2012). Quantifying the Effect of Metformin Treatment and Dose on Glycemic Control. Diabetes Care.

[36] Flory J., Lipska K. (2019). Metformin in 2019. JAMA.

[37] Escribano O., Guillen C., Nevado C., Gomez-Hernandez A., Kahn C.R., Benito M. (2009). Beta-Cell hyperplasia induced by hepatic insulin resistance: Role of a liver-pancreas endocrine axis through insulin receptor A isoform. Diabetes.

[38] Basu R., Barosa C., Jones J., Dube S., Carter R., Basu A., Rizza R.A. (2013). Pathogenesis of Prediabetes: Role of the Liver in Isolated Fasting Hyperglycemia and Combined Fasting and Postprandial Hyperglycemia. J. Clin. Endocrinol. Metab..

[39] Hegarty B.D., Turner N., Cooney G.J., Kraegen E.W. (2009). Insulin resistance and fuel homeostasis: The role of AMP-activated protein kinase. Acta Physiol..

[40] Szendroedi J., Phielix E., Roden M. (2011). The role of mitochondria in insulin resistance and type 2 diabetes mellitus. Nat. Rev. Endocrinol..

[41] El-Mir M.Y., Nogueira V., Fontaine E., Avéret N., Rigoulet M., Leverve X. (2000). Dimethylbiguanide inhibits cell respiration via an indirect effect targeted on the respiratory chain complex I. J. Biol. Chem..

[42] Owen M.R., Doran E., Halestrap A.P. (2000). Evidence that metformin exerts its anti-diabetic effects through inhibition of complex 1 of the mitochondrial respiratory chain. Biochem. J..

[43] Miller R.A., Chu Q., Xie J., Foretz M., Viollet B., Birnbaum M.J. (2013). Biguanides suppress hepatic glucagon signalling by decreasing production of cyclic AMP. Nature.

[44] An H., He L. (2016). Current understanding of metformin effect on the control of hyperglycemia in diabetes. J. Endocrinol..

[45] Malin S.K., Kashyap S.R. (2014). Effects of metformin on weight loss: Potential mechanisms. Curr. Opin. Endocrinol. Diabetes. Obes..

[46] Lupi R., Marchetti P., Giannarelli R., Coppelli A., Tellini C., Del Guerra S., Lorenzetti M., Carmellini M., Mosca F., Navalesi R. (1997). Effects of glibenclamide and metformin (alone or in combination) on insulin release from isolated human pancreatic islets. Acta Diabetol..

[47] Lupi R., Del Guerra S., Fierabracci V., Marselli L., Novelli M., Patane G., Boggi U., Mosca F., Piro S., Del Prato S. (2002). Lipotoxicity in Human Pancreatic Islets and the Protective Effect of Metformin. Diabetes.

[48] Schatz H., Katsilambros N., Nierle C., Pfeiffer E.E. (1972). The effect of biguanides on secretion and biosynthesis of insulin in isolated pancreatic islets of rats. Diabetologia.

[49] Gelin L., Li J., Corbin K.L., Jahan I., Nunemaker C.S. (2018). Metformin Inhibits Mouse Islet Insulin Secretion and Alters Intracellular Calcium in a Concentration-Dependent and Duration-Dependent Manner near the Circulating Range. J. Diabetes Res..

[50] Tucker G.T., Casey C., Phillips P.J., Connor H., Ward J.D., Woods H.F. (1981). Metformin kinetics in healthy subjects and in patients with diabetes mellitus. Br. J. Clin. Pharmacol..

[51] Graham G.G., Punt J., Arora M., Day R.O., Doogue M.P., Duong J.K., Furlong T.J., Greenfield J.R., Greenup L.C., Kirkpatrick C.M. (2011). Clinical pharmacokinetics of metformin. Clin. Pharmacokinet..

[52] Henquin J.-C., Dufrane D., Kerr-Conte J., Nenquin M. (2015). Dynamics of glucose-induced insulin secretion in normal human islets. Am. J. Physiol. Endocrinol. Metab..

[53] Godsland I.F., Jeffs J.A.R., Johnston D.G. (2004). Loss of beta cell function as fasting glucose increases in the non-diabetic range. Diabetologia.

[54] Straub S.G., James R.F., Dunne M.J., Sharp G.W. (1998). Glucose activates both K(ATP) channel-dependent and K(ATP) channel-independent signaling pathways in human islets. Diabetes.

[55] MacDonald M.J., Longacre M.J., Langberg E.-C., Tibell A., Kendrick M.A., Fukao T., Ostenson C.-G. (2009). Decreased levels of metabolic enzymes in pancreatic islets of patients with type 2 diabetes. Diabetologia.

[56] Masini M., Anello M., Bugliani M., Marselli L., Filipponi F., Boggi U., Purrello F., Occhipinti M., Martino L., Marchetti P. (2014). Prevention by metformin of alterations induced by chronic exposure to high glucose in human islet beta cells is associated with preserved ATP/ADP ratio. Diabetes Res. Clin. Pract..

[57] Cen J., Sargsyan E., Forslund A., Bergsten P. (2018). Mechanisms of beneficial effects of metformin on fatty acid-treated human islets. J. Mol. Endocrinol..

[58] Robertson R.P., Harmon J., Tran P.O.T., Poitout V. (2004). Beta-cell glucose toxicity, lipotoxicity, and chronic oxidative stress in type 2 diabetes. Diabetes.

[59] Twig G., Elorza A., Molina A.J.A., Mohamed H., Wikstrom J.D., Walzer G., Stiles L., Haigh S.E., Katz S., Las G. (2008). Fission and selective fusion govern mitochondrial segregation and elimination by autophagy. EMBO J..

[60] Gier B., Krippeit-Drews P., Sheiko T., Aguilar-Bryan L., Bryan J., Düfer M., Drews G. (2009). Suppression of KATP channel activity protects murine pancreatic β cells against oxidative stress. J. Clin. Investig..

[61] Eizirik D.L., Cardozo A.K., Cnop M. (2008). The role for endoplasmic reticulum stress in diabetes mellitus. Endocr. Rev..

[62] Nadanaka S., Okada T., Yoshida H., Mori K. (2007). Role of disulfide bridges formed in the luminal domain of ATF6 in sensing endoplasmic reticulum stress. Mol. Cell. Biol..

[63] Shen J., Chen X., Hendershot L., Prywes R. (2002). ER stress regulation of ATF6 localization by dissociation of BiP/GRP78 binding and unmasking of golgi localization signals. Dev. Cell.

[64] Haze K., Yoshida H., Yanagi H., Yura T., Mori K. (1999). Mammalian transcription factor ATF6 is synthesized as a transmembrane protein and activated by proteolysis in response to endoplasmic reticulum stress. Mol. Biol. Cell.

[65] Marchetti P., Bugliani M., Lupi R., Marselli L., Masini M., Boggi U., Filipponi F., Weir G.C., Eizirik D.L., Cnop M. (2007). The endoplasmic reticulum in pancreatic beta cells of type 2 diabetes patients. Diabetologia.

[66] Oyadomari S., Araki E., Mori M. (2002). Endoplasmic reticulum stress-mediated apoptosis in pancreatic β -cells. Apoptosis.

[67] Kato K., Gong J., Iwama H., Kitanaka A., Tani J., Miyoshi H., Nomura K., Mimura S., Kobayashi M., Aritomo Y. (2012). The Antidiabetic Drug Metformin Inhibits Gastric Cancer Cell Proliferation In Vitro and In Vivo. Mol. Cancer Ther..

[68] Stamateris R.E., Sharma R.B., Hollern D.A., Alonso L.C. (2013). Adaptive beta-cell proliferation increases early in high-fat feeding in mice, concurrent with metabolic changes, with induction of islet cyclin D2 expression. Am. J. Physiol. Endocrinol. Metab..

[69] Sharma R.B., O’Donnell A.C., Stamateris R.E., Ha B., McCloskey K.M., Reynolds P.R., Arvan P., Alonso L.C. (2015). Insulin demand regulates β cell number via the unfolded protein response. J. Clin. Investig..

[70] Wyett G., Gibert Y., Ellis M., Castillo H.A., Kaslin J., Aston-Mourney K. (2018). Metformin, beta-cell development, and novel processes following beta-cell ablation in zebrafish. Endocrine.

[71] Jiang Y., Huang W., Wang J., Xu Z., He J., Lin X., Zhou Z., Zhang J. (2014). Metformin plays a dual role in MIN6 pancreatic β cell function through AMPK-dependent autophagy. Int. J. Biol. Sci..

[72] Livak K.J., Schmittgen T.D. (2001). Analysis of relative gene expression data using real-time quantitative PCR and the 2(-Delta Delta C(T)) Method. Methods.

