# Supplementary material for: Metformin Preserves β-Cell Compensation in Insulin Secretion and Mass Expansion in Prediabetic Nile Rats
Source: Int J Mol Sci. 2021 Jan 3;22(1):421. doi: 10.3390/ijms22010421 (PMC7794750; doi:10.3390/ijms22010421)
Supplement: Supplementary file 1 [file ijms-22-00421-s001.pdf]

International Journal of *Molecular Sciences*

MDPI

Supplementary Table S1: Metabolic profile of NRs treated with metformin

Outcome	Hfib	Chow	Chow+met
Body weight (g)	71.5±1.9	77.0±2.6	78.8±2.3
BMI (kg/m^2)	4.6±0.1	4.8±0.1	4.8±0.1
Weight gain (%)	46±8	44±5	44 ±5
**Fasting state**			
FBG (mmol/L)	2.9±0.3	3.2±0.7	2.5±0.3
Insulin (ng/mL)	0.6±0.5	4.9±1.7*	5.0±1.7*
ISI	1.7±0.2	1.0±0.2*	1.0±0.2
HOMA-IR	2.0±1.5	23.0±11.0*	16.7±6.3
HOMA-B	0.9±0.7	7.6±2.7*	9.1±3.0*
Glucagon (pg/mL)	47.8±36.3	325.7±74.2*	480.6±162.2*
Glucagon/Insulin	0.7±0.5	0.4±0.2	0.7±0.4
**Fed state**			
Glucose (mmol/L)	3.2±0.2	3.3±0.2	3.6±0.2
Insulin (ng/mL)	2.8±0.4	7.0±1.41	11.3±2.3*
Glucagon (pg/mL)	102.1±30.2	89.0±33.7	55.9±23.9
Glucagon/Insulin	0.04±0.01	0.01±0.004	0.02±0.01
GLP-1 (pmol/L)	1.2±0.2	0.7±0.4	0.4±0.1

BMI: body mass index; ISI: insulin sensitivity index; HOMA-IR: homeostatic model assessment of insulin resistance; HOMA-B: homeostatic model assessment of β-cell function. Data were represented as mean ± SEM and analyzed using the Kruskal-Wallis test. *p<0.05 vs age-matched Hfib.

Supplementary Table S2: Antibodies and dilutions used in western blot and immunofluorescent microscopy

Antibody	Source	Dilution
Rabbit Anti-AMPKα	2532, Cell Signaling Technology, Danvers, MA, USA	1:1000
Rabbit Anti-phospho-Thr172-AMPKα	2535, Cell Signaling Technology, Danvers, MA, USA	1:1000
Rabbit Anti- PEP CK	10004943, Cayman Chemical, Ann Arbor, MI, USA	1:200
Rabbit Anti-G6Pase	Sc-25840, Santa Cruz Biotechnology, Dallas, Texas, USA	1:1000

Mouse Anti-Glut2	Sc-518022, Santa Cruz Biotechnology, Dallas, Texas, USA	1:200
Rabbit Anti-IR	3025, Cell Signaling Technology, Danvers, MA, USA	1:500
Rabbit Anti-PGC1α	Ab54481, Abcam, Cambridge, UK	1:1000
Rabbit Anti-PKA C	4782, Cell Signaling Technology, Danvers, MA, USA	1:1000
Rabbit Anti-phospho-Thr197-PKA	4781, Cell Signaling Technology, Danvers, MA, USA	1:1000
Rabbit Anti-Bip	3177, Cell Signaling Technology, Danvers, MA, USA	1:1000
Rabbit Anti-Grp94	2104, Cell Signaling Technology, Danvers, MA, USA	1:1000
Mouse Anti-CHOP	2895, Cell Signaling Technology, Danvers, MA, USA	1:500
Rabbit Anti-caspase3	9665, Cell Signaling Technology, Danvers, MA, USA	1:500
Mouse Anti-CHOP	2895, Cell Signaling Technology, Danvers, MA, USA	1:500
Rabbit Anti-phospho-Ser79-ACC	3661, Cell Signaling Technology, Danvers, MA, USA	1:1000
Guinea Pig Anti-Insulin	A0564, Dako, Burlington, ON, Canada	1:200
Mouse Anti-Glucagon	ab10842, Abcam, Cambridge, UK	1:200
Rabbit Anti-PDI	3501, Cell Signaling Technology, Danvers, MA, USA	1:200
Rabbit Anti-ERp44	3798, Cell Signaling Technology, Danvers, MA, USA	1:200
Rabbit Anti-Ki67	Ab15580, Abcam, Cambridge, UK	1:200

Supplementary Table S3: qPCR primers and annealing temperatures

Gene		Primer	Tm (°C)
Ins	Forward	5’-AAGTGGCACAACTGGAGCTG-3’	58

	Reverse	5'-GATGCTGGTGCAGCACTGA-3'	
IR	Forward	5'-AGACCCGAAGATTTCCGAGAC-3'	58
	Reverse	5'-GAGCCTCGGATGACTGTGAG-3'	
Glut2	Forward	5'- GTCAGAAGACAAGATCACCGGAAC -3'	58
	Reverse	5'- CCTCTTGAGGTGCATTGATCACAC -3'	
Gck	Forward	5'-GAGCTGGTACGACTTGTGCT -3'	58
	Reverse	5'-AACCGCTCCTTGAAGCTCG -3'	
Pdx1	Forward	5'-GCTGGAGCTGGAGAAGGAATTC-3'	58
	Reverse	5'-CTTCATGCGACGGTTTTGGAACC-3'	
MafaA	Forward	5'-AGTTCGAGGTGAAGAAGGAGCC-3'	58
	Reverse	5'-CGCTCATCCAGTACAGATCCTCC-3'	
Pgc1α	Forward	5'-CCAGCCTCTTTGCCCAGAT-3'	58
	Reverse	5'-AGGGCAATCCGTCTTCATCC-3'	
Atf4	Forward	5'-AACATGACCGAGATGAGCTTCCTG-3'	56
	Reverse	5'-AAGTGCTTGGCCACCTCCA-3'	
Atf6	Forward	5'-TGCTCTGGAACAGGGCTC-3'	56
	Reverse	5'-ATGGACACCAGGATCCTCCA-3'	
spliced *Xbp1*	Forward	5'-CTGAGTCCGCAGCAGGT-3'	56
	Reverse	5'-GGTCCAACTTGTCCAGAATGCC-3'	
Xbp1	Forward	5'-AGTCCGCAGCACTCAGACTA-3'	56
	Reverse	5'-GGTCCAACTTGTCCAGAATGCC-3'	
Chop	Forward	5'-GGAGCTGGAAGCCTGGTATGAG-3'	56
	Reverse	5'-TGGTCAGGCGCTCGATTTCC-3'	
Bax	Forward	5'-CAGGGTTTCATCCAGGATCGAGC-3'	56
	Reverse	5'-GCAATCATCCTCTGCAGCTCC -3'	
Bcl-2	Forward	5'-GGATGACTGAGTACCTGAACCGG-3'	56
	Reverse	5'-GTCTTCAGAGACAGCCAGGAG -3'	

β-Actin	Forward	5’-TATCCTGGCCTCACTGTCCA-3’	56
	Reverse	5’-AAGGGTGTAAAACGCAGCTCA-3’	

Supplementary Figure S1

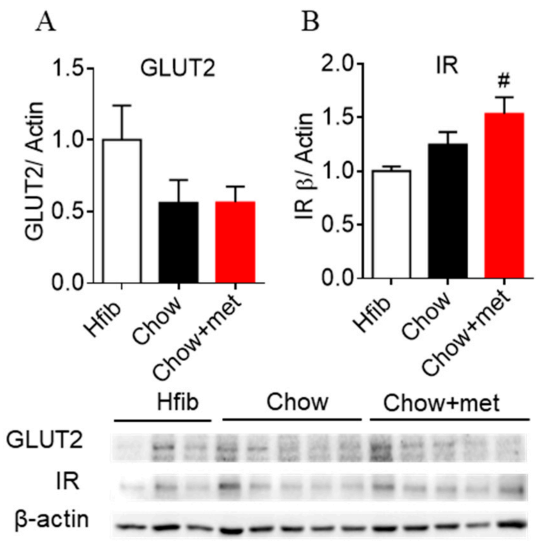

Supplementary Figure S1 Protein abundance of GLUT2 (A) and IR (B) in isolated islets. Data are presented as means + SEM. n=3-5. *indicates p<0.05 vs Hfib, # p<0.05 vs Chow using the Kruskal-Wallis test followed by Dunn's multiple comparison test.

Supplementary Figure S2

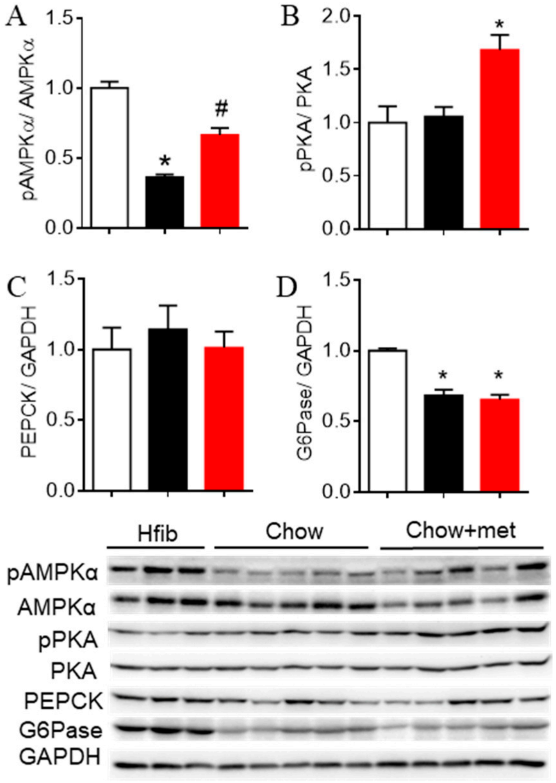

Supplementary Figure S2 Analysis of hepatic AMPK pathway in the fasting condition. A-D quantification of phospho-AMPK to AMPK (A), phospho-PKA to PKA (B), PEPCK (C) and G6Pase (D) relative to GAPDH. Data are presented as means + SEM. n=5-11. *indicates $p<0.05$ vs Hfib, # $p<0.05$ vs Chow using the Kruskal-Wallis test followed by Dunn's multiple comparison test.
